# Short‐term response of a declining woodland bird assemblage to the removal of a despotic competitor

**DOI:** 10.1002/ece3.4016

**Published:** 2018-04-16

**Authors:** Galen Davitt, Kimberly Maute, Richard E. Major, Paul G. McDonald, Martine Maron

**Affiliations:** ^1^ School of Earth and Environmental Sciences The University of Queensland Brisbane QLD Australia; ^2^ Australian Museum Research Institute, Australian Museum Sydney NSW Australia; ^3^ School of Biological Sciences University of Wollongong Wollongong NSW Australia; ^4^ Centre for Behavioural and Physiological Ecology Zoology University of New England Armidale NSW Australia

**Keywords:** biotic homogenization, despotic species, interspecific competition, key threatening process, *Manorina melanocephala*, noisy miner, woodland

## Abstract

Interspecific aggression by the noisy miner (*Manorina melanocephala*), a highly despotic species, is homogenizing woodland avifaunas across eastern Australia. Although a native species, the noisy miner's aggressive exclusion of small birds is a Key Threatening Process under national law. Large‐scale removal of noisy miners has been proposed as a management response to this threat following increases in miner presence due to anthropogenic land use practices. We tested this proposal by experimentally removing noisy miners from eucalypt woodland remnants (16–49 ha), assigned randomly as control (*n* = 12) or treatment (miner removal) sites (*n* = 12). Standardized bird surveys were conducted before and after removal, and generalized linear mixed models were used to investigate the effect of miner removal on bird assemblage metrics. Despite removing 3552 noisy miners in three sessions of systematic shooting, densities of noisy miners remained similarly high in treatment and control sites, even just 14 days after their removal. However, there was evidence of an increase in richness and abundance of small birds in treatment sites compared to controls—an effect we only expected to see if noisy miner densities were drastically reduced. We suggest that miner removal may have reduced the ability of the recolonizing miners to aggressively exclude small birds, even without substantially reducing miner densities, due to the breakdown of social structures that are central to the species' despotic behaviour. However, this effect on small birds is unlikely to persist in the long term. *Synthesis and applications*: Despite evidence from other studies that direct removal of noisy miners can result in rapid and sustained conservation benefit for bird communities at small scales, our findings cast doubt on the potential to scale‐up this management approach. The circumstances under which direct control of noisy miners can be achieved remain unresolved.

## 1 INTRODUCTION

A key mechanism through which landscape change drives shifts in faunal assemblages is the replacement of specialized and fragmentation‐sensitive species by competitive commensal or invasive species. Such shifts often result in an overall reduction in species diversity and the biotic homogenization of the ecosystem (Howes et al., 2014; McKinney & Lockwood, 1999; Tabarelli, Peres, & Melo, 2012). These impacts extend to ecosystem services such as pollination, seed dispersal, soil stability and generation, soil fertility, pest control, and climate regulation (Hooper et al., 2005; Şekercioğlu, Daily, & Ehrlich, 2004), and so identifying how to combat them is an important field of inquiry.

Although invasive species are generally considered the main protagonists of such shifts, native species that benefit from land use change can also expand in distributions or increase in density, leading to ecological impacts no less severe than those of invasive alien species (Bauer, 2012; Haythorpe, Burke, & Sulikowski, 2013). Often these native species that become agents of ecological dysfunction have been favored by anthropogenic disturbance (Bauer, 2012; Hobbs et al., 2006). For example, the brood parasitic brown‐headed cowbird (*Molothrus ater*) has benefited from the expansion of grazing lands and the increase in forest–farmland edges in North America, and poses a threat to several of its hosts such as Bell's vireo (*Vireo bellii*) (Gustafson, Knutson, Niemi, & Friberg, 2002; Kus, 1997). Species that have disproportionately large ecological effects are labeled “keystone” species, or more generally, “strong interactors” (MacArthur, 1972; Menge, Berlow, Blanchette, Navarrete, & Yamada, 1994) and they can be native or introduced. These strong interactors may affect assemblages and ecosystems through a variety of ecological processes, including predation (Menge et al., 1994), habitat transformation (Naiman, Melillo, & Hobbie, 1986), and competition (Piper & Catterall, 2003).

**Figure 1:**
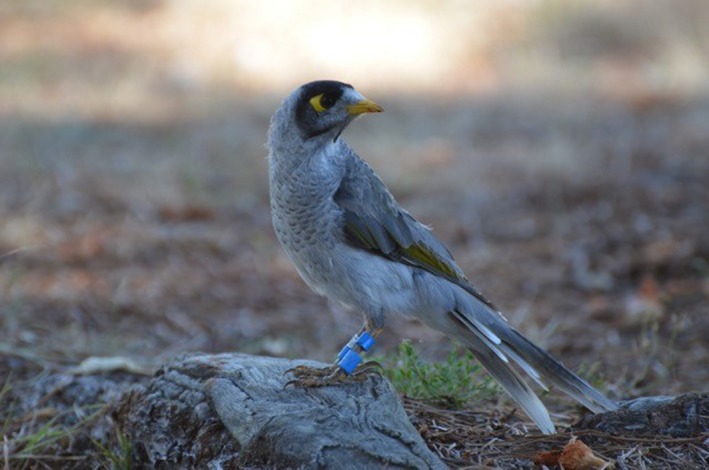
The noisy miner *Manorina melanocephala* is native to woodlands and open forests of eastern Australia

Native *Manorina* honeyeater species are strong competitors within the extensive Australian woodland systems, with bell miners (*Manorina melanophrys*), yellow‐throated miners (*Manorina flavigula*), and noisy miners (*Manorina melanocephala*) implicated in the widespread decline in small woodland birds (Kutt, Vanderduys, Perry, Mathieson, & Eyre, 2015; Leseberg, Lambert, & McDonald, 2015; Maron et al., 2013). The presence of noisy miners (Figure 1) has increased substantially in nine bioregions across eastern Australia (and decreased in none) since 1998 (Maron et al., 2013), and it is likely that the species is more common now than ever before, given the substantially altered landscape and habitat structure throughout these regions (Thomson et al., 2015). Interference competition from the noisy miner in particular is increasingly being recognized as one of the strongest drivers of avian assemblage composition in eastern Australia (Mac Nally & Horrocks, 2002; Maron et al., 2011; Piper & Catterall, 2003; Robertson, Maron, Buckley, & McAlpine, 2013). The species achieves this effect through its despotic habitat selection, whereby it excludes dozens of other bird species from areas of suitable habitat, prompting widespread community‐level shifts across much of eastern Australia's woodland (Clarke & Oldland, 2007; Dow, 1977; Howes & Maron, 2009; Loyn, 2002; Maron et al., 2013; Thomson et al., 2015). Species diversity is reduced and the composition of the avian assemblage is homogenized where noisy miners occur (Howes et al., 2014; Mac Nally, Bowen, Howes, McAlpine, & Maron, 2012).

The noisy miner is a medium‐sized (63 g) passerine whose range extends over 1.3 million km^2^ of eastern Australia (Higgins, Peter, & Steele, 2001). Noisy miners reach peak density in open eucalypt woodland, preferring edges adjacent to agricultural fields and sites with low structural complexity (Campi & MacNally, 2001; Eyre, Maron, Mathieson, & Haseler, 2009; Howes & Maron, 2009; Mac Nally & Horrocks, 2002). The species establishes resident, high‐density, and hyper‐aggressive colonies that exclude almost all smaller and similar‐sized bird species from the areas that they occupy (Maron et al., 2013). Colonies can include several hundred individuals, and the effect of noisy miners on the assemblage is consistent with a threshold effect: a density of >0.6 individuals per ha consistently reduces the richness, abundance, breeding activity, and breeding success of smaller species (<63 g) across eastern Australia (Thomson et al., 2015).

Due to the strong interactive effects that the noisy miner has on avian assemblage structure and ecosystem health over much of eastern Australian, aggressive exclusion by the species has been listed as a Key Threatening Process (KTP) under relevant legislation in New South Wales (NSW Government 2013), Victoria (Flora and Fauna Guarantee 2001), and nationally (Threatened Species Scientific Committee 2013). However, no national threat abatement plan yet exists, and the vast majority of research has focused on characterizing the species' impacts, with few tests of management approaches. Identifying how to manage the impact of noisy miners on already‐declining woodland birds, in a cost‐effective way, is therefore a priority.

When dealing with an undesirable native species, passive management, such as habitat manipulation, is generally more acceptable to the public. However, it is not always the most appropriate or cost‐effective approach for ecosystem management (McAlpine et al., 2016; Scott, Wehtje, & Wehtje, 2001). Landscape restoration in the form of replanting or encouraging regeneration of vegetation to reduce habitat suitability for miners has been found to be of limited benefit in managing the impacts of this species on other woodland birds, at least in the medium term (Mortelliti et al., 2016). On the other hand, small‐scale trial removals of noisy miners in Victoria led to promising conservation outcomes. Grey, Clarke, and Loyn (1997, 1998) found that the removal of noisy miners from seven small (1.6–8 ha) patches of woodland could be done at relatively low cost, and a rapid and dramatic improvement in avian diversity and abundance followed. However, no larger‐scale experiments have yet been conducted to test this potential for active management.

Here, we aimed to test experimentally the short‐term effects of removal of noisy miners from half of a set of 24 woodland patches in central New South Wales, Australia, using a BACI (Before After Control Impact) design. We designed the study to sample a range of landscape and patch‐scale characteristics to determine whether there are particular landscape contexts in which removal is most effective. We present the initial results of this large‐scale field experiment to contribute to the urgently needed body of information about management of this key threatening process.

## 2 MATERIALS AND METHODS

### 2.1 Study area and experimental design

**Figure 2:**
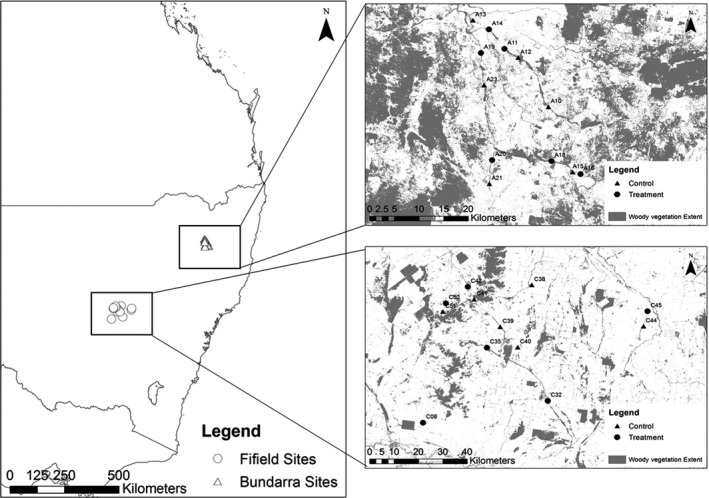
The distribution of Fifield (○) and Bundarra (∆) survey sites in NSW, Australia (left). Inset: distribution of Fifield and Bundarra survey sites with woody vegetation extent (grey) and nonwoody vegetation extent (white) (NSW Government 2015) (ESRI 2014)

The study was conducted in 24 open eucalypt woodland remnants within two distinct biogeographical regions of NSW: New England Tablelands Bioregion (hereafter, “Bundarra” sites, *n* = 12) and South Western Slopes Bioregion (hereafter, “Fifield” sites, *n* = 12) (Figure 2). Both landscapes were highly fragmented but they differed in woodland cover, with only 17% woody vegetation cover remaining in the South Western Slopes Bioregion and 49% cover in the New England Tablelands Bioregion (OEH 2016). The intervening agricultural land was used primarily for cattle and sheep grazing in the New England Tablelands Bioregion, and for a combination of sheep grazing and the production of wheat and other cereals in the Southwest Slopes Bioregion (NPWS 2003).

In each region, six sites were randomly assigned to treatment (= noisy miner removal) and six to control groups. All 24 sites were located in a section of a Travelling Stock Route (TSR), a network of reserves originally retained to aid in the movement and agistment of livestock in areas adjacent to roads. Each site was at least 50 m wide at its narrowest point and was between 16 and 49 ha in extent. Most were approximately rectangular in shape. Sites needed to be large enough to contain two 400 m × 50 m transects that were separated from each other by 400 m, yet small enough so that removal of noisy miners was logistically feasible. At each site, the presence of noisy miners was confirmed by the species' response to 5 min of broadcast of noisy miner vocalizations.

### 2.2 Noisy miner removal

On each of three removal sessions, a trained, licensed shooter supervised by two experienced ornithologists attempted to remove all noisy miners from treatment sites, using a 12‐gauge shotgun loaded with size 8 shot (approx. 410 pellets per cartridge), fired from a distance of 10–30 m. Initially, noisy miners were attracted to the shooter by broadcasting recorded ground alarm calls (chur calls: Holt et al., 2017) at intervals of ~100 m along the length of study site. After the initial pass, the three personnel spread out across the width of the site and walked the length of the site 1–3 times locating and shooting any remaining birds. A total of 3,552 birds were removed across three removal sessions (approx. 100 birds per cull per site) and on the last day of each removal, it was estimated that only between 1 and 10 birds remained at each site.

During each removal session, each treatment site was visited on 2 days for a minimum of 8 hr in total. The first, second, and third removals were carried out at the Fifield sites from 19–24 August, 2015; 12–17 September, 2015; and 12–19 April, 2016, respectively. The first, second, and third removal sessions were carried out at the Bundarra sites from 14–20 November 2015; 4–10 December, 2015; and 3–10 May, 2016, respectively (Supporting Information).

### 2.3 Bird surveys

Bird surveys were conducted at each of three stages: preremoval, postremoval 1 (immediately after the first removal session), and postremoval 2 (3–4 weeks after the final removal session). At each stage, birds were surveyed in two 400 m × 50 m belt transects at each site, with two repeat surveys per transect on separate days, yielding four samples per site at each stage. The preremoval surveys were done between 2 and 19 days before treatment, the postremoval 1 survey between 4 and 17 days after the first removal, and the postremoval 2 surveys were done between 21 and 29 days after the final removal.

All surveys were conducted by a single observer (KM) who traversed the centre of the belt transect, recording all birds seen or heard within 25 m each side of the 400 m transect line, during a 20‐min period. This duration was selected to generate data that were compatible with the Birdlife Australia standard survey of 2 ha in 20 min (Barrett, Silcocks, Barry, Cunningham, & Poulter, 2003). However, because longer survey durations tend to improve detection rates and richness estimates (Watson, 2004), a second survey was conducted traversing the transect in the opposite direction, immediately following each survey in the forward direction. Data from these forward and backward surveys were pooled for analyses as described below. Repeat surveys of the two transects in each site were conducted approximately 4 days after the initial surveys, so that each site had two repeats of each of two transects in each site in each survey period. All bird surveys were conducted in the morning (sunrise to 3 hr after sunrise) or afternoon (2 hr before sunset to sunset), and the sequence of site visitation was rotated so that all sites received at least one morning survey in each period. Surveys were confined to days without rainfall or strong winds to ensure that there was adequate bird detectability.

### 2.4 Environmental data

We measured three environmental variables: patch width, buffer vegetation cover, and shrub density, all of which are likely to influence noisy miner presence (Bennett, Clarke, Thomson, & Mac Nally, 2015; Clarke & Oldland, 2007; Howes & Maron, 2009; Robertson et al., 2013) and also, potentially, colonization rates by miners and small birds (Clarke & Schedvin, 1997; Robertson et al., 2013). Patch width was correlated with patch area. Buffer vegetation cover was based on the extent of woody vegetation (woodland, forest, and paddock trees) within a 1.1 km radius of each site, based on the interpatch crossing distances for birds in this system reviewed by Doerr, Doerr, and Davies (2010). It was estimated visually from 2014 aerial imagery using the Buffer command in ARC Map 10.2.2 (ESRI 2014), and sites were assigned to three categories: low (<10%), medium (10%–20%), and high (>30%). Shrub density was defined as the percentage cover of foliage less than 2 m in height, based on the average of ten 10‐m^2^ quadrats located along each transect, and was visually estimated in the field between April and June 2016.

### 2.5 Data analyses

The abundance of noisy miners and four bird community metrics were calculated for each transect using the bird survey data, and compared between three stages of treatment (preremoval, postremoval 1, and postremoval 2; variable referred to hereinafter as ‘stage’). The four community metrics were (1) mean abundance of all species, (2) mean abundance of small‐bodied woodland bird species, (3) species richness of all species, and (4) species richness of small‐bodied woodland bird species, with all metrics excluding noisy miners. Small‐bodied bird species were defined as those smaller than the noisy miner (<63 g) (Thomson et al., 2015). Species richness was calculated as the sum of species seen or heard during both the forward and backward surveys of each site at each stage of treatment, while mean abundance was calculated as the average number of individuals detected across both forward and backward surveys.

Generalized linear mixed models (GLMMs) were used to investigate the effect of noisy miner removal on the bird assemblage metrics (Table 1). Mixed‐effects models were used as they allow us to account explicitly for the repeat samples and the spatially nested structure of the data (multiple samples within transects within sites) (Zuur, Ieno, Walker, Saveliev, & Smith, 2009). The bird response variables were modelled as a function of treatment (noisy miner removal vs. control), stage (preremoval, postremoval 1, or postremoval 2), and the interaction of main interest: treatment × stage. In addition, region (Bundarra or Fifield), buffer vegetation cover, shrub density, and patch width were included as fixed effects. Given the small number of replicates (24), potential three‐way interactions between treatment, stage, and environmental variables were not included. All models included a random effect of transect nested within site. All explanatory variables were inspected graphically for colinearity and model assumptions were tested by examining the dispersion of residuals.

**Table 1:** Summary of frequency of variable inclusion in models within 2 AICc of the most parsimonious model for each of the five bird response variables, and improvement in AICc over a null model including only the random factor

Response	No. models <2∆AICc	% of best models in which variable included							AICc	
		Treatment	Stage	Treatment*Stage	Region	Shrub density	Buffer veg cover	Patch width	Best model	Null model
Noisy miner abundance	9	56	67	0	100	67	0	78	1555.2	1571.3
All bird abundance	9	44	0	0	78	44	100	33	2127.0	2143.7
All bird species richness	3	100	100	67	100	33	0	100	1342.0	1371.2
Small bird abundance	2	100	100	100	100	50	0	100	1675.9	1711.0
Small bird species richness	1	100	100	100	100	100	100	100	816.3	865.3

An information theoretic approach in a multimodel framework was used to investigate the relative importance of predictor variables (Burnham & Anderson, 2002). For each of the four bird response variables, alternative models with different combinations of predictor variables and interactions were produced using an all‐subsets approach, including the null (intercept‐only) model. The models were compared based on their Akaike weights calculated from AICc values (Akaike's Information Criterion corrected for small sample size) using the MuMIn package 1.15.6 in R (Barton, 2016). Models within ~2 AICc units of the most parsimonious model were considered to have similar levels of empirical support (Burnham & Anderson, 2002). We used model averaging to derive averaged coefficient estimates and 95% confidence intervals for each predictor, allowing us to identify the relative importance of the predictors.

## 3 RESULTS

A total of 93 species was recorded from 2,119 bird records within the survey transects (Supporting Information). The noisy miner was the most abundant species throughout the surveys, with observed numbers of miners being higher in Bundarra compared to Fifield. This was also reflected in the number of individuals removed, with more birds removed from Bundarra sites (*n* = 2,340) than Fifield sites (*n* = 1,212).

### 3.1 Noisy miner abundance

**Figure 3:**
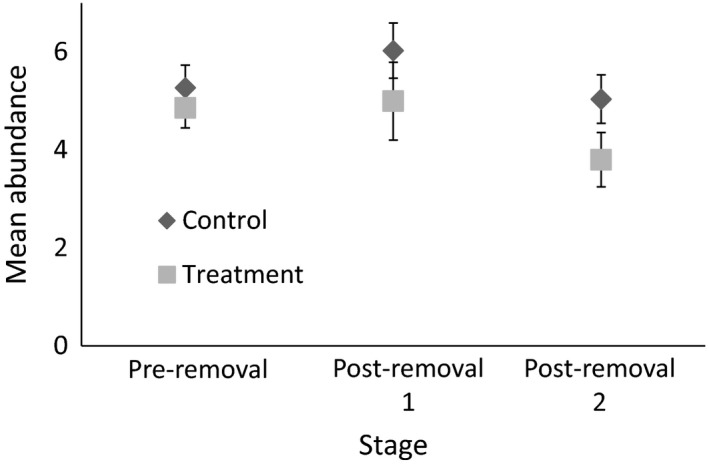
Mean (±*SE*) abundance of noisy miners per survey at each stage in control and treatment sites

There was high model uncertainty with nine models within two AICc values of the best model of noisy miner abundance. Only region was reliably included in the best models (Table 1), but 95% confidence intervals on all other averaged coefficients included zero (Table 2). None of the best models included the interaction between stage and treatment (Table 1), meaning there was no support for our expectation that noisy miner removal led to changes in noisy miner abundance during bird surveys. Thus, despite removing more than 3,500 individuals, any reduction in noisy miner abundance following removal was small by the time our follow‐up surveys were conducted (Figure 3).

**Table 2:** Averaged coefficients and 95% confidence interval for models of noisy miner abundance

Variable	Estimate	±95% CI
Intercept	6.605	2.064
Treatment (Removal)	−0.508	1.262
Stage (Post 1)	0.305	0.888
Stage (Post 2)	−0.413	0.975
Treatment*Stage (Post 1)	−0.020	0.393
Treatment*Stage (Post 2)	−0.026	0.387
Region (Fifield)	−2.673	1.210
Shrub density	0.077	0.142
Buffer vegetation cover (high)	0.048	0.815
Buffer vegetation cover (med)	0.202	0.972
Patch width	−0.002	0.003

### 3.2 All bird species

Only buffer vegetation cover was reliably included in the best models of the mean abundance of all bird species, with a positive effect (Table 1), but the 95% confidence intervals of the averaged coefficient included zero. Model uncertainty was high, with nine models within 2 AICc values of the best model. There was moderate support for an effect of region, with Fifield sites having on average 2.4 fewer individuals per survey than Bundarra sites (Table 3). The stage × treatment interaction was included in none of the best‐performing models, suggesting no effect of noisy miner removal on bird abundance when calculated for all other species combined.

**Table 3:** Averaged coefficients and 95% confidence interval for models of all bird abundance and species richness. Bold indicates CI does not include zero

Variable	Abundance		Richness	
	Estimate	±95% CI	Estimate	95% CI
Intercept	12.147	3.471	1.804	0.244
Treatment (Removal)	0.487	2.248	0.071	0.190
Stage (Post 1)	−0.052	1.623	0.037	0.140
Stage (Post 2)	−0.629	2.722	−**0.284**	**0.207**
Treatment*Stage (Post 1)	0.121	1.585	0.018	0.169
Treatment*Stage (Post 2)	0.284	2.629	0.147	0.312
Region (Fifield)	−1.728	2.990	**−0.252**	**0.148**
Shrub density	0.068	0.216	−0.003	0.012
Buffer vegetation cover (high)	**6.431**	**3.332**	0.008	0.082
Buffer vegetation cover (med)	1.680	2.590	−0.001	0.058
Patch width	0.001	0.003	<0.001	<0.001

**Figure 4:**
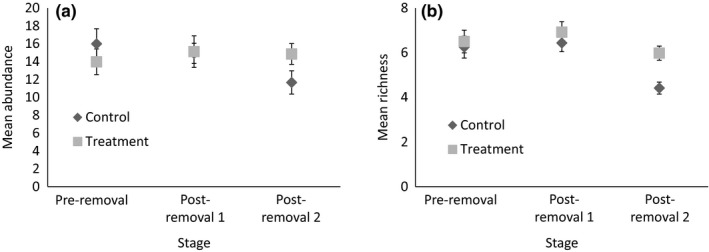
(a) Mean (±*SE*) abundance and (b) species richness of all birds (excluding noisy miners) at each stage for control and treatment sites

Two of the three models within 2 AICc values of the best model of total species richness included the interaction between stage and treatment (Table 1). This suggests that noisy miner removal may have had a small positive effect on species richness, despite the negligible effect of culling on noisy miner density, although the 95% confidence intervals around the averaged coefficient included zero (Table 3). Species richness in both control and treatment sites remained steady following the first removal, but after the final removal, species richness was lower—although this was most pronounced in control sites, with an average of 1.6 fewer species per survey than treatment sites (Figure 4). The best models of species richness all included the variables region and patch width, with the Fifield region having fewer species than Bundarra; however, these effects were highly uncertain (Table 3).

### 3.3 Small bird responses

**Figure 5:**
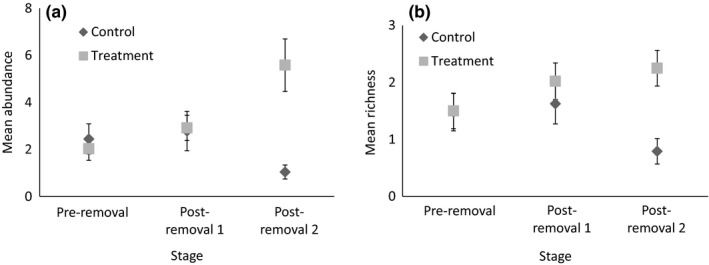
(a) Mean (±*SE*) abundance of small birds (<63 g), and (b) species richness of small birds at each stage for control and treatment sites

Both the best models for abundance and the single best model of species richness of small birds included the interaction between stage and treatment (Table 1), suggesting removal of noisy miners had a positive effect on small bird richness and abundance. This effect was evident after the second removal for both abundance and richness (Figure 5; Table 4). The mean abundance of small birds in treatment sites more than doubled between preremoval and postremoval 2 stages, while the number of birds in control sites remained similar (Figure 5, Table 4).

**Table 4:** Averaged coefficients and 95% confidence interval for models of small bird abundance and species richness. Bold indicates CI does not include zero

Variable	Abundance		Richness	
	Estimate	95% CI	Estimate	95% CI
Intercept	2.261	2.227	−0.351	0.850
Treatment (Removal)	−0.203	1.943	0.617	0.622
Stage (Post 1)	0.333	1.594	0.094	0.323
Stage (Post 2)	−1.406	1.593	−0.625	0.396
Treatment*Stage (Post 1)	0.552	2.253	0.204	0.455
Treatment*Stage (Post 2)	**4.958**	**2.253**	**1.031**	**0.496**
Region (Fifield)	−**2.981**	**1.494**	−1.335	0.560
Shrub density	−0.034	0.126	−**0.077**	**0.061**
Buffer vegetation cover (high)	0.162	1.114	−0.012	0.362
Buffer vegetation cover (med)	−0.044	0.720	0.065	0.381
Patch width	**0.005**	**0.003**	**0.003**	**0.002**

Small bird richness and abundance were positively influenced by patch width, and the Fifield region had a lower mean abundance of small birds (Tables 1 and 4).

## 4 DISCUSSION

Despite removing 3,552 noisy miners (~10 birds/ha), there was no statistical support for a reduction in noisy miner abundance at treatment sites, indicating that the species could recolonize rapidly. However, even though the treatment failed to reduce noisy miner density beyond a few days, surprisingly it led to at least a short‐term increase in the abundance and species richness of small birds in particular. This effect was not as pronounced as would be expected if noisy miners were successfully extirpated, but the fact that it was evident despite no substantial reduction in the number of miners present is perplexing and may reflect how a perturbation to the noisy miner's social structure alters the effectiveness of its interspecific aggression.

### 4.1 Effectiveness of noisy miner removal

Remarkably, despite the removal of an average of almost 300 birds per site, there was no statistical support for noisy miner removal as an important predictor of noisy miner abundance. Over the course of three removal sessions, the removal of noisy miners from Bundarra and Fifield sites led to only a slight (22%) reduction in the observed number of noisy miners from 4.9 to 3.8 birds per transect in treatment sites. This result is inconsistent with the findings of Grey et al. (1997, 1998), who reported that the removal of noisy miners from experimental sites led to an initial decrease in mean density of noisy miners by 35%–71%. They also identified that noisy miners appeared to temporarily reinvade treatment sites during the removal exercise, without permanently establishing in the “vacant area”. In contrast, similar numbers of birds were removed in this study 1 month and 7 months after the first removal.

The lack of a treatment effect despite the removal treatment might conceivably have been because noisy miners flew out of treated sites in the short‐term during shooting, and so the cumulative effect of multiple removals was required to achieve measurable reductions in miner density. However, observations during removal sessions suggested that few birds flew outside of the remnant to paddock trees, and most were intercepted during repeat passes of the remnant. Furthermore, given the sheer number of individuals removed during the study, it is most likely that the majority of resident birds were killed during the removal sessions, and those encountered during subsequent surveys were colonists from elsewhere in the landscape.

### 4.2 Effect of noisy miner removal on the woodland bird community

Despite the statistically negligible reduction in noisy miner numbers at treatment sites, these sites did show an increase in abundance and species richness of small birds—the group most susceptible to noisy miner aggression (Mac Nally, McAlpine, Possingham, & Maron, 2014; Thomson et al., 2015). These increases in small birds in treatment sites were only apparent after the third round of removals. After these removals, there may have been sufficient reduction in noisy miner numbers to have reduced harassment and territorial aggression toward small birds. However, as we have described, the reduction in miner abundance was small, and the final survey density of 1.9 miners per ha recorded during the bird surveys described herein was still well above the threshold of 0.6 individuals per ha that has been found to result in pronounced negative impacts on smaller bird species (Thomson et al., 2015).

We propose that the increase in small birds may instead have been a result of disruption of noisy miner social structure, with residents being replaced by newcomers that were more engaged with behaviors associated with colonization rather than interspecific aggression and eviction from the new colony boundaries. Noisy miners have an extremely complex social system that is reliant upon helpers exhibiting social behavior and cooperation across a range of contexts from cooperative breeding to mobbing potential competitors or predators (Dow & Whitmore, 1990; Farrow, Doohan, & McDonald, 2017; Kennedy, Evans, & McDonald, 2009). Given this, the considerable upheaval of colony removal and subsequent recolonization may have impacted the newly occupying miners' ability to adequately defend these areas, hence the increase in small bird diversity. If this did occur, then this disruption to noisy miner group dynamics likely provided a temporary opportunity to small birds to exploit the resources in these sites.

This increase in small bird abundance was only detected after the final removal, in autumn, perhaps due to a seasonal effect associated with a temporal increase in mixed foraging flock activity, which is greatest during autumn/winter in temperate climates (Bell, 1980). Immediate movement of small birds into treatment sites may be more likely to occur at a time when there is a greater number of birds travelling through the landscape than during the breeding season when birds are more sedentary. However, our study was conducted over a short period, and the benefits of noisy miner culling are unlikely to be long lasting if colonizing individuals or groups are able to rebuild social structures and effectively exclude small birds within a few seasons. Continued survey effort is necessary to determine the longevity of the benefits for small bird species.

### 4.3 Conservation implications

Although the results of this study are intriguing, with a detectable effect of noisy miner removal on small birds, the removal did not substantially reduce densities of noisy miners as expected based on smaller‐scale removals (Debus, 2008; Grey et al., 1997, 1998). Our results also conflict with expectations based on the work of Thomson et al. (2015) and Mac Nally et al. (2012), who showed that a threshold negative effect of noisy miners on the richness and abundance of small bird species occurs reliably at densities of just 0.6 and 0.8 individuals per ha, respectively. The densities of noisy miners in this study remained much greater than those thresholds, yet increases in small birds were detected in treatment sites. This suggests that the disruption to the birds' social structure could result in a short‐term positive response by small birds, possibly due to less efficient or less aggressive behavior of new colonists.

Similar to findings by earlier noisy miner removals (Debus, 2008; Grey et al., 1997, 1998), the recolonization of degraded sites by woodland birds occurred without restoration of the understory. Although the effect is expected to be temporary, even this response suggests that there may be short‐term benefits associated with attempts to control noisy miners near nesting sites for highly sensitive species, such as the critically endangered regent honeyeater (*Xanthomyza phrygia*). Such attempts may be beneficial in reducing harassment of nesting honeyeaters even if reductions in noisy miners are not evident. However, future experimentation across a wider range of landscape and vegetation structures would help to determine the circumstances under which benefits to small birds can be achieved by noisy miner removal.

While continued survey effort is needed to identify the duration of this effect, we show clearly that noisy miner recolonization can be immediate and include a dramatically larger number of individuals than expected based on past research (Grey et al., 1997, 1998). The extremely high densities of noisy miners recorded in this study also highlight the species' ability to use and disperse through largely cleared landscapes that surround remnant woodlands, increasing the difficulty of removing sufficient birds from target habitat.

Importantly, the scale at which the removals occurred was substantially larger than previously explored (Debus, 2008; Grey et al., 1997, 1998) (16–49 ha, compared with 3–15 ha in previous removals). The limited effectiveness of the removals compared with those conducted at smaller scales is intriguing and counterintuitive. One speculative explanation is that removing a single coterie or subunit of a colony does not disrupt overall colony territoriality, and the remainder of the colony prevents “new” miners from establishing in the vacated region, effectively creating a localized reduction in miner density until filled by within‐colony recruitment. By removing the entire colony, we may have left the vacated area ‘undefended’ and inadvertently facilitated rapid recolonization of the site in landscapes densely populated by noisy miner colonies in potentially less suitable habitat.

At this stage, we are not able to draw firm conclusions about the mechanisms producing these complex results and a key issue to resolve is the provenance of noisy miners that occupy sites following removal. It is important to conclusively resolve whether newly arrived miner colonists originate from one intact adjacent colony moving, for example, or represent multiple combined dispersal events of small groups of birds from a nearby colony “budding off” or dispersing, both strategies seen in the congeneric bell miner (Dare et al., 2008).

This study demonstrated at best limited conservation utility of noisy miner removal in the circumstances trialed, at the scale of tens of hectares. Several management trials in different regions of Australia are underway, and it is critical that these actions are appropriately monitored to allow sound evaluation of their effectiveness. Further experimentation in different regions and at different spatial scales is required to determine the circumstances under which noisy miner control may be both cost and environmentally effective in liberating woodland bird habitat.

## CONFLICT OF INTEREST

None declared.

## AUTHORS' CONTRIBUTIONS

RM, PM, GD, and MM conceived the ideas and designed methodology; GD, KM, RM, and PM collected the data; GD analyzed the data; GD led the writing of the manuscript. All authors contributed critically to the drafts and gave final approval for publication.

## DATA ACCESSIBILITY

Data available from the Dryad Digital Repository: https://doi.org/10.5061/dryad.42355kp.
