# Supplementary material for: Short‐term response of a declining woodland bird assemblage to the removal of a despotic competitor
Source: Ecol Evol. 2018 Apr 16;8(10):4771–80. doi: 10.1002/ece3.4016 (PMC5980597; doi:10.1002/ece3.4016)
Supplement: Supplementary file 1 [file ECE3-8-4771-s001.docx]

**Supporting Information:** Treatment Summaries for Fifield and Bundarra regions
