# Supplementary material for: Short‐term response of a declining woodland bird assemblage to the removal of a despotic competitor
Source: Ecol Evol. 2018 Apr 16;8(10):4771–80. doi: 10.1002/ece3.4016 (PMC5980597; doi:10.1002/ece3.4016)
Supplement: Supplementary file 2 [file ECE3-8-4771-s002.docx]

**Supporting Information:** Species detected on < 25 m woodland transects throughout the duration of the study

| **Common Name** | **Genus species** | **Order** |
| --- | --- | --- |
| Grey Teal | *Anas gracilis* | Anseriformes |
| Pacific Black Duck | *Anas superciliosa* | Anseriformes |
| Australian Wood Duck | *Chenonetta jubata* | Anseriformes |
| Crested Pigeon | *Ocyphaps lophotes* | Coloumbiformes |
| Common Bronzewing | *Phaps chalcoptera* | Coloumbiformes |
| Pallid Cuckoo | *Cacomantis pallidus* | Cuculiformes |
| Channel-billed Cuckoo | *Scythrops novaehollandiae* | Cuculiformes |
| Tawny Frogmouth | *Podargus strigoides* | Podargiformes |
| White-faced Heron | *Egretta novaehollandiae* | Pelecaniformes |
| Brown Goshawk | *Accipiter fasciatus* | Accipitriformes |
| Spotted Harrier | *Circus assimilis* | Accipitriformes |
| Laughing Kookaburra | *Dacelo novaeguineae* | Coraciiformes |
| Dollarbird | *Eurystomus orientalis* | Coraciiformes |
| Sacred Kingfisher | *Todiramphus sanctus* | Coraciiformes |
| Brown Falcon | *Falco berigora* | Falconiformes |
| Nankeen Kestrel | *Falco cenchroides* | Falconiformes |
| Sulphur-crested Cockatoo | *Cacatua galerita* | Psittaciformes |
| Major Mitchell's Cockatoo | *Cacatua leadbeateri* | Psittaciformes |
| Galah | *Cacatua roseicapilla* | Psittaciformes |
| Little Corella | *Cacatua sanguinea* | Psittaciformes |
| Australian Ringneck | *Barnardius zonarius* | Psitaciformes |
| King Parrot | *Alisterus scapularis* | Psittaciformes |
| Red-winged Parrot | *Aprosmictus erythropterus* | Psittaciformes |
| Musk Lorikeet | *Glossopsitta concinna* | Psittaciformes |
| Little Lorikeet | *Glossopsitta pusilla* | Psittaciformes |
| Blue Bonnet | *Northiella haematogaster* | Psittaciformes |
| Crimson Rosella | *Platycercus elegans* | Psittaciformes |
| Eastern Rosella | *Platycercus eximius* | Psittaciformes |
| Superb Parrot | *Polytelis swainsonii* | Psittaciformes |
| Red-rumped Parrot | *Psephotus haematonotus* | Psittaciformes |
| Rainbow Lorikeet | *Trichoglossus moluccanus* | Psittaciformes |
| White-throated Treecreeper | *Cormobates leucophaeus* | Passeriformes |
| Brown Treecreeper | *Climacteris picumnus* | Passeriformes |
| Superb Fairy-wren | *Malurus cyaneus* | Passeriformes |
| Brown Honeyeater | *Lichmera indistincta* | Passeriformes |
| Scarlet Honeyeater | *Myzomela sanguinolenta* | Passeriformes |
| Striped Honeyeater | *Plectorhyncha lanceolata* | Passeriformes |
| Little Friarbird | *Philemon citreogularis* | Passeriformes |
| Noisy Friarbird | *Philemon corniculatus* | Passeriformes |
| Blue-faced Honeyeater | *Entomyzon cyanotis* | Passeriformes |
| Brown-headed Honeyeater | *Melithreptus brevirostris* | Passeriformes |
| Spiny-cheeked Honeyeater | *Acanthagenys rufogularis* | Passeriformes |
| Red Wattlebird | *Anthochaera carnunculata* | Passeriformes |
| Yellow-faced Honeyeater | *Lichenostomus chrysops* | Passeriformes |
| Fuscous Honeyeater | *Lichenostomus fuscus* | Passeriformes |
| White-plumed Honeyeater | *Lichenostomus penicillatus* | Passeriformes |
| Yellow-throated Miner | *Manorina flavigula* | Passeriformes |
| Noisy Miner | *Manorina melanocephala* | Passeriformes |
| Spotted Pardalote | *Pardalotus punctatus* | Passeriformes |
| Striated Pardalote | *Pardalotus striatus* | Passeriformes |
| Western Gerygone | *Gerygone fusca* | Passeriformes |
| White-throated Gerygone | *Gerygone olivacea* | Passeriformes |
| Weebill | *Smicrornis brevirostris* | Passeriformes |
| Speckled Warbler | *Chthonicola sagittata* | Passeriformes |
| White-browed Scrubwren | *Sericornis frontalis* | Passeriformes |
| Inland Thornbill | *Acanthiza apicalis* | Passeriformes |
| Yellow-rumped Thornbill | *Acanthiza chrysorrhoa* | Passeriformes |
| Striated Thornbill | *Acanthiza lineata* | Passeriformes |
| Yellow Thornbill | *Acanthiza nana* | Passeriformes |
| Buff-rumped Thornbill | *Acanthiza reguloides* | Passeriformes |
| Chestnut-rumped Thornbill | *Acanthiza uropygialis* | Passeriformes |
| Grey-crowned Babbler | *Pomatostomus temporalis* | Passeriformes |
| Varied Sittella | *Daphoenositta chrysoptera* | Passeriformes |
| Black-faced Cuckoo-shrike | *Coracina novaehollandiae* | Passeriformes |
| White-bellied Cuckoo-shrike | *Coracina papuensis* | Passeriformes |
| White-winged Triller | *Lalage sueurii* | Passeriformes |
| Golden Whistler | *Pachycephala pectoralis* | Passeriformes |
| Rufous Whistler | *Pachycephala rufiventris* | Passeriformes |
| Grey Shrike-thrush | *Colluricincla harmonica* | Passeriformes |
| Crested Shrike-tit | *Falcunculus frontatus* | Passeriformes |
| Olive-backed Oriole | *Oriolus sagittatus* | Passeriformes |
| Pied Currawong | *Strepera graculina* | Passeriformes |
| Pied Butcherbird | *Cracticus nigrogularis* | Passeriformes |
| Grey Butcherbird | *Cracticus torquatus* | Passeriformes |
| Australian Magpie | *Gymnorhina tibicen* | Passeriformes |
| Dusky Woodswallow | *Artamus cyanopterus* | Passeriformes |
| White-browed woodswallow | *Artamus superciliosus* | Passeriformes |
| Grey Fantail | *Rhipidura fuliginosa* | Passeriformes |
| Willie Wagtail | *Rhipidura leucophrys* | Passeriformes |
| Australian Raven | *Corvus coronoides* | Passeriformes |
| Little Raven | *Corvus mellori* | Passeriformes |
| Restless Flycatcher | *Myiagra inquieta* | Passeriformes |
| Leaden Flycatcher | *Myiagra rubecula* | Passeriformes |
| Magpie-lark | *Grallina cyanoleuca* | Passeriformes |
| White-winged Chough | *Corcorax melanorhamphos* | Passeriformes |
| Apostlebird | *Struthidea cinerea* | Passeriformes |
| Jacky Winter | *Microeca fascinans* | Passeriformes |
| Mistletoebird | *Dicaeum hirundinaceum* | Passeriformes |
| Double-barred Finch | *Taeniopygia bichenovii* | Passeriformes |
| Rufous Songlark | *Cincloramphus mathewsi* | Passeriformes |
| Welcome Swallow | *Hirundo neoxena* | Passeriformes |
| Common Starling | *Sturnus vulgaris* | Passeriformes |
